# Complete Genome Sequences of Thermus thermophilus Strains AA2-20 and AA2-29, Isolated from Arima Onsen in Japan

**DOI:** 10.1128/MRA.00820-19

**Published:** 2019-08-01

**Authors:** Kentaro Miyazaki, Natsuki Tomariguchi

**Affiliations:** a Bioproduction Research Institute, National Institute of Advanced Industrial Science and Technology (AIST), Tsukuba, Ibaraki, Japan; b Department of Computational Biology and Medical Sciences, Graduate School of Frontier Sciences, The University of Tokyo, Kashiwa, Chiba, Japan; c Faculty of Life Sciences, Toyo University, Itakura, Gunma, Japan; Georgia Institute of Technology

## Abstract

We isolated halophilic and thermophilic Thermus thermophilus strains AA2-20 and AA2-29 from nonvolcanic, oceanic Arima Onsen (hot spring) in Japan. Here, we report the complete genome sequences of these organisms to gain insights into halophilicity.

## ANNOUNCEMENT

*Thermus thermophilus* was first isolated by Oshima and Imahori from Mine Onsen (hot spring) in Japan (1). The bacterium grows optimally at 70 to 75°C, and its thermophilic nature aids the study of biomacromolecules, such as enzymes (2–7) and ribosomes (8, 9), whose functional investigations are often limited due to the lack of stability. We isolated T. thermophilus strains AA2-20 and AA2-29 from nonvolcanic, oceanic Arima Onsen in Kobe, Japan (10). The environmental sample was spread over TYSS (1% [wt/vol] tryptone, 0.5% [wt/vol] yeast extract, and 4% [wt/vol] sea salts [Sigma]) agar plates. After overnight incubation at 65°C, two colonies with red or yellow pigmentation (named AA2-20 and AA2-29, respectively) were isolated. DNA sequencing analysis of the rRNA gene suggested that the two strains belong to T. thermophilus (99% identity to the T. thermophilus HB8 gene). Both the strains displayed limited growth in low-salt media, such as Lennox LB (1% [wt/vol] tryptone, 0.5% [wt/vol] yeast extract, and 0.5% [wt/vol] NaCl) broth, thereby suggesting their halophilic nature.

To prepare genomic DNA, the two strains were grown in TYSS broth at 65°C until saturation. Genomic DNA was extracted using the Nexttec 1-step DNA isolation kit for bacteria (Nexttec Biotechnologie) according to the manufacturer’s instructions.

For long-read sequencing, the extracted genomic DNA was passed through a Circulomics short-read eliminator kit to remove short fragments. Sequencing was performed using a GridION X5 system (Oxford Nanopore Technologies [ONT]); the library was constructed from unfragmented genomic DNA (1.0 μg) using a ligation sequencing kit (ONT) and applied to a FLO-MIN106 R9.41 flow cell (ONT). The long-read sequences, which were base called using Guppy v.3.0.3, further generated 278,671 reads (1,550 Mb) with an average length of 5,563 bases during a 24-h run time (the data are associated with quality-filtered reads with average phred quality values of >8.0, obtained using NanoFilt v.2.3.0 [11]). The longest read had 91,228 bases.

For short-read sequencing, the extracted genomic DNA was used for library preparation with the Nextera DNA library preparation kit (Illumina). Prepared libraries were subjected to 100-bp paired-end sequencing on the Illumina HiSeq 2500 platform. Adapters and low-quality sequencing data were trimmed using fastp v.0.14.1 (12), and 6.9 million paired-end reads (660 Mb) with an average length of 96 bases were obtained.

Hybrid assembly of long-read and short-read data was conducted using Unicycler v.0.4.7 (13), followed by a final polishing with Pilon v.1.23 (14), which resulted in the production of a single circular chromosome and a single circular plasmid. Automated annotation was performed using DFAST v.1.1.0 (15). Default parameters were used for all software unless otherwise noted.

The genome statistics and genomic features are listed in Table 1. A JSpecies analysis (16) revealed that the genomic sequences of AA2-20 and AA2-29 were nearly identical (97.2% average nucleotide identity) with no large gaps or rearrangement. Additionally, these sequences showed high average nucleotide identity (89.6%) with the genomic sequence of T. thermophilus JL-18 (GenBank accession number NC_017587), which was isolated from freshwater hot springs in Great Basin National Park, USA. The strains AA2-20 and AA2-29 differed from JL-18 with respect to large rearrangements in their sequences, including inversions and indels.

**TABLE 1:** Genome statistics and genomic features of T. thermophilus isolates

Strain name	Chromosome or plasmid	Length (bp)	GC content (%)	No. of CDSs^*a*^	No. of rRNAs	No. of tRNAs	DDBJ accession no.
AA2-20	Chromosome	1,928,002	69.2	2,045	6	51	AP019792
	Plasmid	219,630	68.9	258	0	0	AP019793
AA2-29	Chromosome	19,290,112	69.2	2,042	6	51	AP019794
	Plasmid	215,278	69.1	252	0	0	AP019795

a CDSs, coding DNA sequences.

### Data availability.

The accession numbers for the complete genome sequences of T. thermophilus strains AA2-20 and AA2-29 are listed in Table 1. Raw sequencing data have been deposited in the DDBJ SRA database under the accession number DRA008626 (BioProject number PRJDB7414, BioSample numbers SAMD00177813 [AA2-20] and SAMD00177814 [AA2-29]). The T. thermophilus strains AA2-20 and AA2-29 have been deposited in the RIKEN Bioresource Center, JCM, under the accession numbers JCM 33047 and JCM 33048, respectively.
